# Gene Localization and Functional Validation of *GmPDH1* in Soybean Against Cyst Nematode Race 4

**DOI:** 10.3390/plants14121877

**Published:** 2025-06-19

**Authors:** Yuehua Dai, Yue Zhang, Chuhui Li, Kun Wan, Yan Chen, Mengen Nie, Haiping Zhang

**Affiliations:** 1 College of Agriculture, Shanxi Agricultural University, Taiyuan 030031, China; dyh122551@163.com (Y.D.); zhangyue980906@163.com (Y.Z.); 18235505461@163.com (C.L.); a18790391973@163.com (K.W.); 2 Center for Agricultural Genetic Resources Research, Shanxi Agricultural University, Taiyuan 030031, China; chenyan0869@126.com (Y.C.); 15035686051@163.com (M.N.)

**Keywords:** soybean, soybean cyst nematode, PDH1, CRISPR/Cas9

## Abstract

To identify the key genes conferring resistance to soybean cyst nematode race 4 (SCN4, *Heterodera glycines*), this study utilized 280 recombinant inbred lines (RILs) derived from the resistant cultivar Huipizhiheidou (HPD) and the susceptible cultivar Jindou23 (JD23). Through phenotypic characterization and a genome-wide association study (GWAS), a genomic region (Gm18:1,223,546–1,782,241) on chromosome 18 was mapped, yielding 14 candidate genes. *GmPDH1* was validated as a critical resistance gene using reverse transcription quantitative PCR (RT-qPCR) and Kompetitive Allele Specific PCR (KASP) marker M0526. RT-qPCR revealed that *GmPDH1* expression in HPD roots was upregulated 9 days post-inoculation with SCN4 compared to uninoculated controls. KASP genotyping showed that marker M0526 efficiently distinguished between resistant and susceptible plants in natural populations: 71.05% of the resistant accessions exhibited resistant or moderately resistant genotypes, whereas 81.03% of the susceptible accessions showed susceptible or highly susceptible genotypes. Functional validation demonstrated that overexpression of *GmPDH1* significantly enhanced SCN4 resistance in the susceptible cultivars JD23 and Jack, whereas CRISPR/Cas9-mediated knockout of *GmPDH1* in HPD attenuated its resistance. This study confirmed *GmPDH1* as a key gene governing SCN4 resistance and developed an efficient molecular marker, M0526, providing theoretical insights and technical tools for dissecting nematode resistance mechanisms and advancing soybean disease-resistant breeding.

## 1. Introduction

Soybean (*Glycine max* L. Merr.) is a critical oilseed crop that plays a vital role in global food and animal feed production. Its cultivation is frequently compromised by abiotic stresses, with soybean cyst nematode (SCN, *Heterodera glycines*) being a predominant biotic constraint on soybean yield worldwide, causing annual economic losses of approximately USD157 billion [1,2]. SCN exhibits extensive distribution across major soybean-producing regions, particularly in the United States and China [3,4]. The pathogenic variability among SCN populations has led to their classification into distinct physiological races. The predominant races in the United States include race 3 (HG type7/PA3), race 14 (HG1.3.6.7), and race 4 (HG type1.2.3.5.7) [5]. In China, the major identified races are Race1 (HG type2.5.7/TN7), race 2 (HG type1.2.5.7/TN22), race 3 (HG type7/PA3), and race 4 (HG type1.2.3.5.7), which are predominantly distributed across the Huang-Huai and Northeast production regions.

The SCN life cycle involves sexual reproduction, progressing through four juvenile stages (J1–J4), with the infective J2 larvae penetrating soybean roots to establish feeding sites (syncytia) through cell wall dissolution [6]. Sexual differentiation occurs at the J4 stage, with postmortem female bodies forming hardened cysts containing eggs that persist in the soil for 3–4 years under unfavorable conditions [7].

Integrated management strategies, including crop rotation, biological control, and cultivation of resistant cultivars, are cost-effective approaches for nematode population control [8,9]. The exploration of disease-resistant gene resources can accelerate the cultivation of disease-resistant varieties. Current resistance breeding primarily utilizes genetic resources from the Peking and PI88788 genotypes, with *Rhg1* (chromosome 18) and *Rhg4* (chromosome 8) recognized as well-studied resistance loci [10]. Peking harbors both Rhg1-a and *Rhg4* alleles, whereas PI88788 possesses *Rhg1-b* [11,12]. The *Rhg1-b* locus contains three resistance-associated genes within a 31-kb region: *Glyma.18g022400* (*GmAAT*), *Glyma.18g022500* (*α*-SNAP, *GmSNAP18*), and *Glyma.18g022700* (*GmWI12*) [13,14]. Copy number variations of these genes collectively mediate SCN resistance. The mechanism of action of α-SNAP encoded by the *Glyma.18g022500* gene has been elucidated. The α-SNAP encoded by *Rhg1* with different copy numbers in various soybean varieties exhibits amino acid polymorphism, thereby affecting resistance. As a conserved protein involved in vesicle transport, α-SNAP binds to N-ethylmaleimide-sensitive fusion protein (NSF) to form a complex, which mediates the cycling of soluble NSF attachment protein receptors and thus regulates cellular vesicle transport [15,16]. When nematodes infect the roots, α-SNAP specifically accumulates at the feeding sites of SCN and indirectly affects the intracellular vesicle transport mechanism through interaction with NSF. In addition to influencing resistance through copy number, the resistance conferred by *Rhg1* is also affected by genetic sequence variations of the gene. A minor resistance gene *Glyma.11g234500* (*GmSNAP11*) homologous to α-SNAP on chromosome 11 enhances the resistance of Peking-type soybeans to SCN [17].

The *Rhg4* locus, initially identified in Peking cultivars, synergistically interacts with *Rhg1-a* to confer resistance. This locus contains four genes encoding adenosylhomocysteinases (*Glyma.08g108800*), serine hydroxymethyltransferase (*SHMT08*, *Glyma.08g108900*), proprotein convertase, and NAD-dependent epimerase/dehydratase [18,19]. Among these, the disease resistance mechanism of the *SHMT* gene has been reported. *SHMT08*-mediated perturbation of one-carbon folate metabolism induces folate deficiency, triggering hypersensitive cell death at nematode feeding sites [18,19]. Beyond these canonical resistance genes, *GmPAL* enhances lignin biosynthesis by activating phenylpropanoid metabolism during nematode infection [20].

*GmPDH1* encodes the PDH1 enzyme, which is involved in the synthesis of L-tyrosine (Tyr). Tyr is synthesized from chorismate, the final product of the shikimate pathway. Chorismate is converted into prephenate by chorismate mutase (CM; EC5.4.99.5). Prephenate is transaminated into arogenate by prephenate aminotransferase (PPA-AT; EC2.6.1.7), which is then dehydrogenated into Tyr by arogenate dehydrogenase (TyrAa, abbreviated as ADH; EC1.3.1.78). This is referred to as the ADH pathway and is commonly found in plants. Another pathway for Tyr synthesis is the PDH pathway, which is commonly observed in microorganisms. Prephenate is first converted into 4-hydroxyphenylpyruvate (HPP) by prephenate dehydrogenase (TyrAp, abbreviated as PDH; EC1.3.1.13) and then transaminated into Tyr by a tyrosine aminotransferase [21,22,23,24,25,26]. In addition to these typical patterns, there are exceptions. Leguminous plants possess both PDH and ADH activity [27]. ADH and PDH belong to the Tyr A protein family and are key regulatory enzymes involved in Tyr biosynthesis. The ADH enzyme is located at the branch point between the biosynthesis of L-phenylalanine (Phe) and Tyr and is often competitively inhibited by Tyr. It also competes for arogenate, a substrate for Phe biosynthesis [28,29,30]. When an adequate amount of Tyr is present, other aromatic amino acids such as Phe can be effectively produced [28,31]. Unlike ADH, which synthesizes Tyr within the plastid, soybean PDH is localized outside the plastid, has higher activity than ADH, and is insensitive to the inhibition of Tyr and its intermediates [27]. This may contribute to the increased Tyr yield in some leguminous plants [32]. In addition, the direct synthesis of Tyr outside the plastid provided by the PDH pathway bypasses the ADH pathway within the plastid, thus escaping the competition for Phe precursors in the plastid, promoting the biosynthesis of Phe and increasing the lignin content [33]. Lignin and its metabolic processes are involved in the nematode resistance response in soybeans [34].

**Figure 1:**
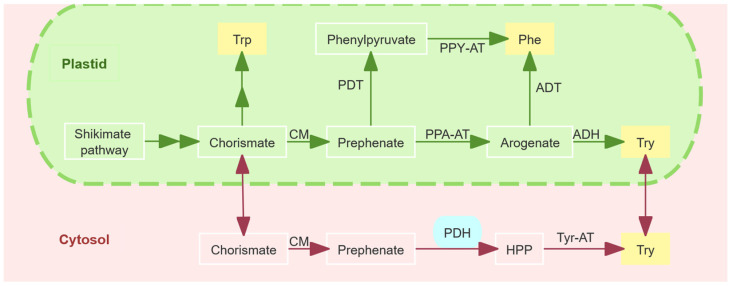
Tyr biosynthesis and associated pathways in legumes. legumes possess a Tyr-insensitive, cytosolic PDH enzyme (blue).

Recent studies have challenged the hypothesis that the PDH pathway compensates for the ADH pathway, indicating that the plastid-localized ADH pathway is the primary route for Tyr biosynthesis in legumes and cannot be compensated for by the cytoplasmic PDH pathway (Figure 1) [27]. However, under stress conditions, PDH enzymes facilitate the production of specialized metabolites derived from Tyr or its synthetic intermediate HPP [35]. Tyr and its intermediate HPP serve as precursors of various functional metabolites in plants, including antioxidants (tocopherols), allelochemicals (L-DOPA), electron transport molecules (plastoquinones), and structural components (lignin). For example, tocopherols (vitamin E) are synthesized via both the HPP pathway (mediated by PDH enzymes from prephenate) and the Tyr metabolic pathway (via Tyr-AT transamination), and their levels are positively correlated with Tyr/HPP concentrations [36,37,38,39]. L-DOPA, an allelochemical in legumes like broad beans, is considered to inhibit weeds and nematodes, and has been shown to be involved in inducing PAL activity and lignin synthesis. However, whether L-DOPA can inhibit or promote PAL activity is still controversial [40,41,42,43,44,45,46].

Previous studies have shown that *GmPDH1* is a key gene in the extraplastidic Tyr synthesis pathway of soybean, and PDH enzymes can respond to high-light stress by promoting the synthesis of specialized metabolites; however, the function of *GmPDH1* in soybean resistance to cyst nematodes has not been clarified [35]. Preliminary genotyping in this study showed that only 34.4% of the plants carrying both major resistance loci (*Rhg1* and *Rhg4*) exhibited resistant/moderately resistant phenotypes, whereas those carrying only *Rhg1* or *Rhg4* were predominantly susceptible. This suggests the presence of additional resistance determinants in the resistant Huipizhiheidou (HPD) cultivar. Therefore, this study aimed to identify novel resistance loci through a genome-wide association study (GWAS), validate *GmPDH1* as a key gene, and elucidate its mechanism of action in SCN4 resistance.

## 2. Results

### 2.1. Phenotypic Profiling and Candidate Gene Identification in Soybean in Response to SCN4 Infestation

In 2021 and 2022, to check disease resistance of the recombinant inbred lines (RIL) population, they were artificially inoculated and identified. Owing to force majeure factors during the planting process, some plants died. Finally, the data for 264 and 233 plants in 2021 and 2022, respectively, were obtained. In the environments of the 2 years, with Lee68 as the control, the female index (FI) of each line was calculated (Table S1). Under the stress of SCN4, the average FIs of the roots of the female parent Jindou23 (JD23) in the two environments were 63.82 and 85.28 respectively, indicating high susceptibility. The average FIs of the male parent HPD were 20.28 and 16.51 respectively, showing moderate resistance, and the coefficients of variation were 40.85% and 45.51% respectively, indicating great differences in disease resistance within the population. The variation ranges of the FIs in the two environments were 2.13–177.28 and 4.59–216.64 respectively. The normal distribution diagrams of FIs conformed to the typical characteristics of the inheritance of quantitative traits and were suitable for GWAS.

**Figure 2:**
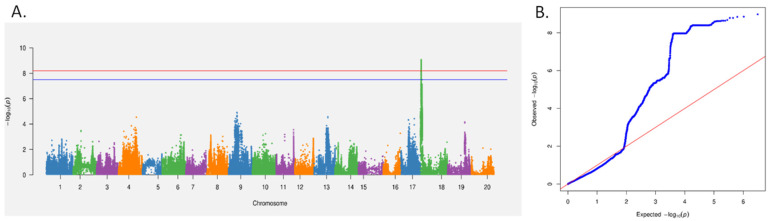
Chromosome 18-centric association analysis of SCN4 resistance. (**A**): Regional Manhattan plot highlighting a 0.56-Mb association hotspot. Blue line denotes SNPs with −log10 (p) ≥ 7.5. Red line denotes SNPs with −log10 (p) ≥ 8.2. (**B**): Chromosome 18-specific Q–Q plot showing observed (blue) vs. expected (red) *p*-value distributions.

High-quality single nucleotide polymorphisms (SNP) loci were selected for further analysis. Combined with the phenotypic data of relevant traits, the mixed linear model (MLM) in EMMAX software (emmax-intel-binary-20120210) was used for GWAS analysis. The threshold standard for SNP loci significantly associated with the traits was −log10(p) ≥ 7.5. Through GWAS, 1062 significant SNPs related to resistance against SCN4 were identified. These 1062 significant SNPs were distributed on chromosomes 1, 11, and 18. Among them, 612 SNP loci were identified in the Gm18:1,223,546–1,782,241 intervals (Figure 2A,B). Genes with missense mutations in the coding sequence (CDS) region were listed as candidate genes. The results showed 14 candidate genes on chromosome 18. Based on the annotation information of genes in the SoyBase database (https://www.soybase.org/ (accessed on 1 March 2023)), this locus was analyzed to identify candidate genes related to resistance against SCN4 (Table S2). Among them, *Glyma.18g022500* encodes the α—SNAP protein. This gene interferes with the function of the NSF and vesicle transport in soybeans by encoding an α—SNAP variant protein, thereby achieving resistance regulation [47].

### 2.2. Developmental Stage-Specific Nematode Quantification in Resistant vs. Susceptible Cultivars

A comparative analysis of SCN developmental progression between resistant (HPD) and susceptible (JD23) soybean cultivars revealed distinct colonization patterns (Table S3). At 6 days post-inoculation (dpi), both genotypes showed J2 penetration, with resistant roots containing 364 J2 larvae/plant compared to 877 in susceptible roots. The susceptible plants exhibited peak J2 populations at this stage. By 10 dpi, the J2 counts increased to 395 (resistant) and 505 (susceptible), suggesting partial J3 transition initiation. J2 populations subsequently declined by 14 dpi, with concurrent J3 accumulation (resistant: 58; susceptible: 132). Developmental divergence became pronounced at 18 dpi, where resistant roots exclusively contained 18 J4 females, whereas susceptible roots harbored both J4 females (24) and males (12). J4 larvae were undetectable in the resistant roots after 22 dpi, whereas the susceptible roots developed 60 mature cysts at 26 dpi. This complete suppression of cyst formation in HPD suggests active resistance mechanisms that disrupt nematode sexual maturation and life cycle completion.

### 2.3. The Expression Patterns of Candidate Genes During SCN4 Infection

To explore the role of the candidate genes in soybean resistance to cyst nematodes and their expression patterns at different infection stages, specific primers were designed based on specific segments of different genes. RT-qPCR was used to analyze the expression of soybean genes under SCN stress. According to the differences in the developmental dynamics of nematodes between resistant and susceptible varieties, a period of 3–12 days is critical for the development of nematodes from J2 to J3 in resistant and susceptible soybeans. Therefore, samples were collected every 3 days to observe the expression characteristics of the candidate genes at different time points.

**Figure 3:**
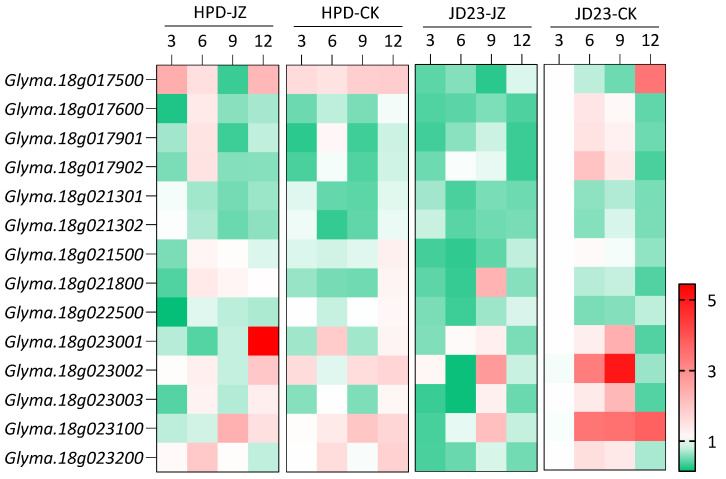
The expression levels of 14 candidate genes were analyzed in in HPD and JD23. HPD-JZ and JD23-JZ represent inoculated HPD and JD23 samples, respectively; HPD-CK and JD23-CK represent uninoculated control groups of HPD and JD23, respectively. 3, 6, 9 and 12 dpi mean that samples were taken at 3, 6, 9 and 12 days after inoculation. Red indicates upregulated relative expression, while green indicates downregulated relative expression.

**Figure 4:**
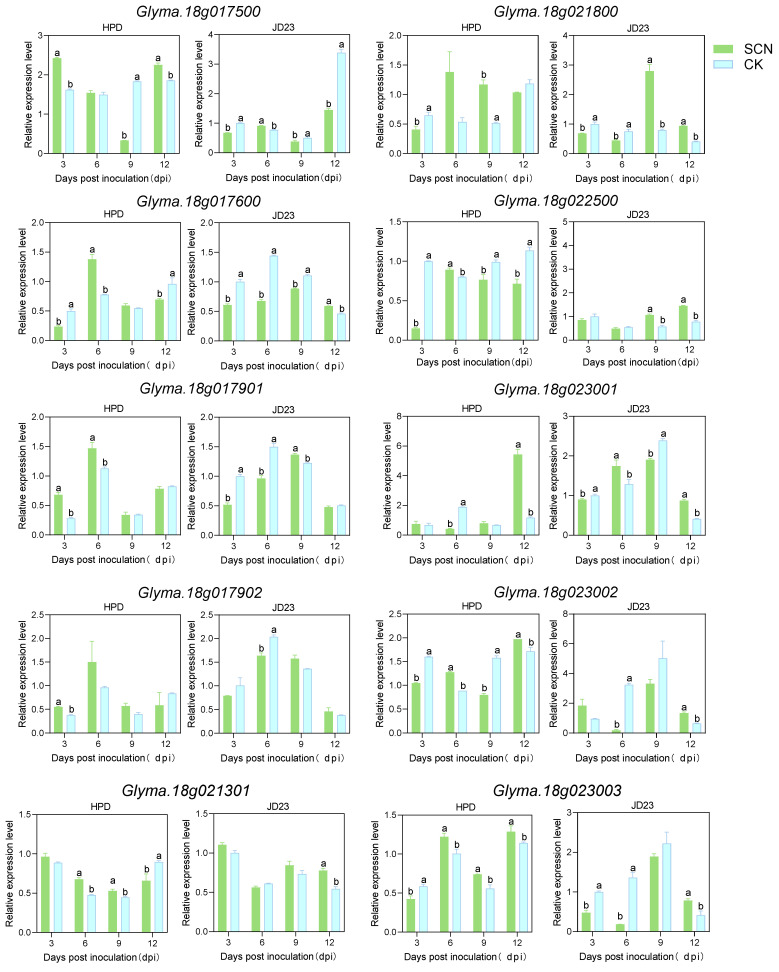

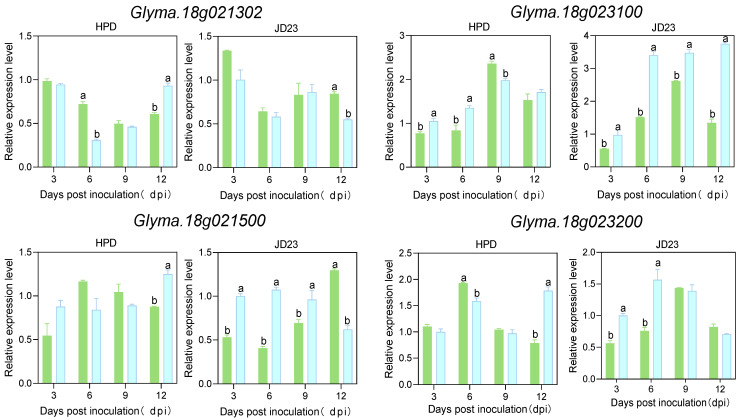
RT-qPCR analysis of the transcriptional level of 14 candidate genes in SCN-resistant HPD and SCN-susceptible JD23 soybeans after SCN4 infection. 3, 6, 9 and 12 dpi mean that samples were taken at 3, 6, 9 and 12 days after inoculation. Transcriptional-level changes of 14 candidate genes were represented by the relative expression-level upregulated/downregulated folds as compared to the control (CK). Transgene expression was normalized using *GmActin* gene and calibrated using the same sample under control conditions. Data shown represent the means ± SD of three independent experiments, with each experiment consisting of three technical replicates. ANOVA was used for statistical analysis. Different lowercase letters above columns represent significant difference among samples at *p* < 0.05.

Comparative RT-qPCR profiling of candidate genes in SCN-infected soybean roots revealed four temporally distinct expression modules associated with the resistance mechanisms (Figure 3). In the resistant cultivar HPD, *Glyma.18g017500* exhibited biphasic induction, with 50% (3 dpi) and 21% (12 dpi) upregulation compared to mock controls, whereas the susceptible cultivar JD23 showed 33% (3 dpi) and 57% (12 dpi) expression reductions. This pattern aligns with the dual defense roles in early J2 penetration inhibition and late J4 maturation blockade. Sustained activation of *Glyma.18g017901* in resistant roots (1.4-fold at 3 dpi) contrasted sharply with progressive suppression in susceptible plants (≤60%), suggesting its involvement in multi-stage defense coordination. A gene cluster (*Glyma.18g017600/Glyma.18g021800/Glyma.18g023200*) displayed transient induction peaks at 6 dpi (0.2–1.6-fold) in resistant lines, temporally coinciding with J2 molting initiation, while being suppressed in susceptible counterparts (≤60%). Delayed activation of *Glyma.18g023100* at 9 dpi (20%) in resistant roots was correlated with disrupted J3 sexual differentiation preceding complete suppression of cyst formation. These temporally coordinated transcriptional responses demonstrate that SCN4 resistance involves stage-specific gene modules targeting discrete developmental transitions: early J2 penetration (3 dpi), molting (6 dpi), and sexual maturation (9–12 dpi), through dynamic expression reprogramming (Figure 4).

However, *Glyma.18g021302*, *Glyma.18g023001*, and *Glyma.18g022500* showed expression patterns opposite to those of most of the above-mentioned genes, especially *Glyma.18g022500*. At 9 and 12 dpi, the relative expression of this gene was downregulated in HPD, whereas it was significantly upregulated in JD23. Combined with the reported disease resistance mechanism of this gene, these results suggest that *Glyma.18g022500* has a different mechanism from that of protein function changes caused by base variation, and it regulates disease resistance through differences in gene copy number [13,48].

### 2.4. Kompetitive Allele-Specific PCR (KASP) Marker Development for the SCN Resistance-Associated GmPDH1 Locus

**Figure 5:**
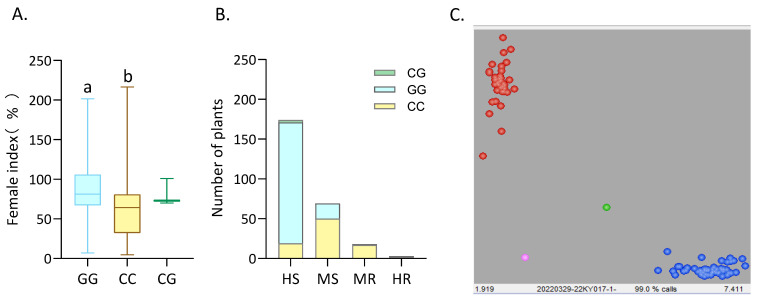
Effect of M0526 on SCN4 resistance, derived from a comparison based on a panel of 264 Chinese soybean entries. (**A**): Reactions of race4 to different combinations of SNP marker alleles for 264 RIL populations. CC indicates the resistant genotype; GG indicates the susceptible genotype; CG indicates the heterozygous genotype. Boxes show median. Whiskers extend to minimum and maximum of the data. The different letters above the boxes represent different significant levels at *p* < 0.05. (**B**): Distribution of different allelic variants of M0526 in resistant and susceptible lines for 264 RIL populations. The four resistance and susceptibility grades divided by the FI include HS, MS, MR and HR. HS: highly susceptible plants; MS: moderately susceptible plants; MR: moderately resistant plants; HR: highly resistant plants. CC indicates plants with resistant genotype; GG indicates plants with susceptible genotype; CG indicates plants with heterozygous genotype. (**C**): Genotyping results of SCN4 resistance by KASP in 96 natural populations. The scatter plot with axes x and y represents allelic discrimination of resistant or susceptible genotypes. The red, green, and blue dots represent the resistant homozygous, heterozygous, and susceptible homozygous, respectively.

We performed a precision genotyping test on a biparental population of F14-derived RILs from HPD × JD23. A randomized complete block design was employed for three replicates of 264 RILs along with both parental lines, and each line was genotyped using M0526 markers. Phenotyping was performed 25 dpi by quantifying the number of cysts on the roots of individual plants. It showed a significant correlation between the FI and genotypes of M0526 with single factor analysis in SPSS (v22.0, IBM corporation, Armonk, NY, USA). Association analyses were conducted on 264 identified lines (Figure 5A,B): 88 lines carried the M0526-CC genotype; 173 carried the M0526-GG genotype; and three carried the M0526-CG genotype. Among the 240 highly and moderately susceptible lines, 171 carried the susceptible genotype M0526-GG, accounting for 71.25% of the total. Among the 21 highly and moderately resistant lines, 19 carried the resistant genotype M0526-CC, accounting for 90.47%. All the three lines with the heterozygous genotype M0526-CG were highly susceptible. The identification efficiencies for resistant and susceptible lines were 90.47% and 71.25%, respectively. It was preliminarily determined that this KASP marker could be used to screen for disease-resistant soybean plants.

To test the specificity and practicality of this marker, 96 natural population lines were used to verify M0526. Except for one disease-resistant line with no data, the genotyping data for marker M0526 in the natural population are shown in Figure 5C. The genotypes of 71.05% of the disease-resistant lines showed resistance or moderate resistance (27 of 38 samples), and the genotypes of 81.03% of the disease-susceptible lines showed susceptibility or high susceptibility (47 of 58 samples). The SNP marker M0526 can be used quickly and efficiently to identify the resistance of soybeans to SCN4 of soybean cyst nematode with a prediction accuracy of 71–90% for resistant and susceptible lines.

### 2.5. Structural and Promoter Cis-Element Profiling of GmPDH1

**Figure 6:**
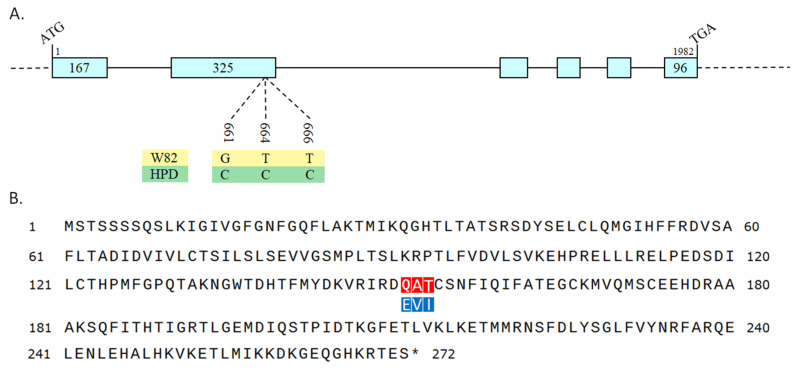
Gene sequence alignment results. (**A**): *GmPDH1* CDS cloning and sequence alignment. There are three missense mutations among HPD and JD23. (**B**): Comparison of the predicted GmPDH1 protein sequences among HPD and JD23 with the amino acid differences (E151Q, V152A, and I153T). Red and blue represent HPD and JD23, respectively, in (**B**); *, After sequence alignment, the same sequences have been merged, and only different sequences are displayed.

The genetic information of *Glyma.18g023100* was queried and compared online using SoyBase (https://www.soybase.org/ (accessed on 1 August 2023)) and NCBI (https://www.ncbi.nlm.nih.gov/ (accessed on 1 August 2023)). *Glyma.18g023100*, also known as *GmPDH1*, encodes the PDH, which belongs to the prephenate/atrogenate dehydrogenase family of proteins and is involved in regulating Tyr synthesis. In addition, by predicting the protein sequences based on the nucleotide differences between resistant and susceptible varieties in the GWAS, three amino acid mutations (E151Q, V152A, and I153T) were identified (Figure 6), which may lead to changes in disease resistance functions.

Cis-regulatory element profiling of the 2000 bp promoter region via PlantCARE (https://bioinformatics.psb.ugent.be/webtools/plantcare/html/ (accessed on 1 August 2023)) uncovered multiple stress-responsive motifs, including MeJA-responsive elements (CGTCA-motif), MYB-binding sites (MBS), anaerobic-induction elements (ARE), light-responsive elements (G-box), gibberellin-responsive elements (P-box), abscisic-acid-responsive elements (ABRE), and defense/stress-responsive elements (TC-rich repeats) (Table S4). The presence of a CAT box motif suggests potential meristem-specific regulation of expression.

### 2.6. Physicochemical Characterization and Tertiary Structure Prediction of the GmPDH1 Protein

Physicochemical analysis using ProtParam (https://web.expasy.org/protparam/ (accessed on 1 August 2023)) revealed *GmPDH1* to be a hydrophilic protein (average GRAVY: −0.226) with a molecular weight of 30.6 kDa and an isoelectric point of 6.55. The 271-amino acid sequence exhibited stability (instability index: 37.85) and aliphatic dominance (instability index: 81.29). Leucine (10.0%), serine (8.9%), and threonine (8.9%) constituted the major residues with a balanced charge distribution (31 basic vs. 33 acidic residues). Secondary structure prediction identified α-helix (50.18%) and random coil (37.64%) as dominant conformations (Table S5). Extended half-lives (>10 h in *Escherichia coli*, >20 h in yeast, and 30 h in mammals) suggest robust protein stability across systems.

### 2.7. Transgenic Soybean Overexpressing GmPDH1 Conferred Further Resistance to SCN4

**Figure 7:**
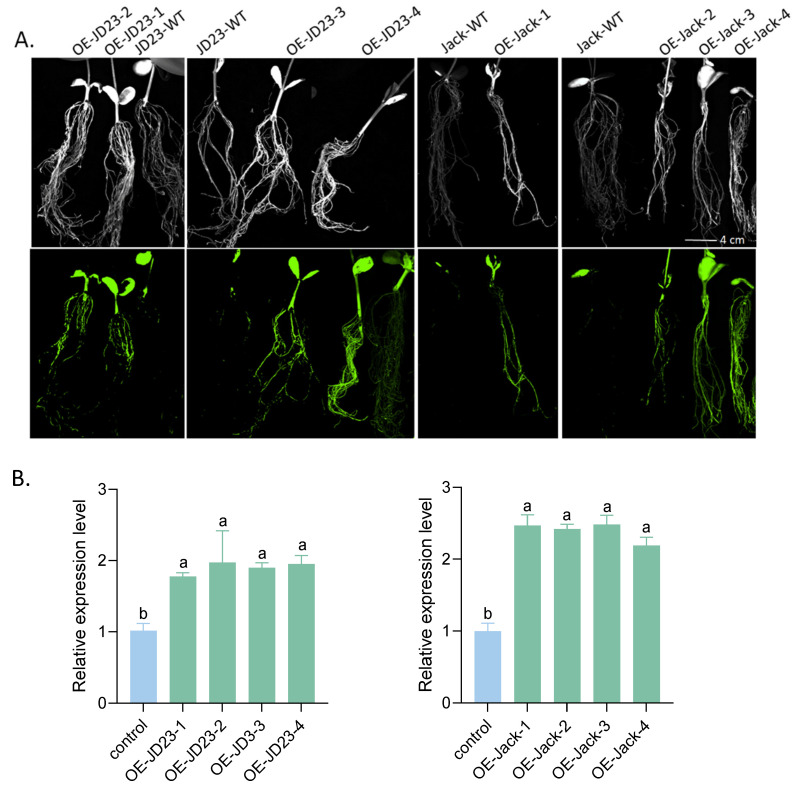
Functional validation of *GmPDH1* in resistance to SCN using expressing vector pCAMBIA1302. (**A**): Composite soybean plants with transgenic GFP-positive hairy roots were selected under fluorescent light using wild-type hairy roots (WT) as controls. Representative images are shown. No gross phenotypic differences were observed in *GmPDH1* expressing roots of either JD23 or Jack. Pictures were taken just before transplanting and nematode inoculation. (**B**): Relative expression levels in overexpressed susceptible lines OE-JD23 and OE-Jack of *GmPDH1* by RT-qPCR analysis. Transgene expression was normalized using *GmActin* gene and calibrated using the same sample under control conditions. Data shown represent the means ± SD of three independent experiments, with each experiment consisting of three technical replicates. ANOVA was used for statistical analysis. Different lowercase letters above columns represent significant difference among samples at *p* < 0.05.

**Figure 8:**
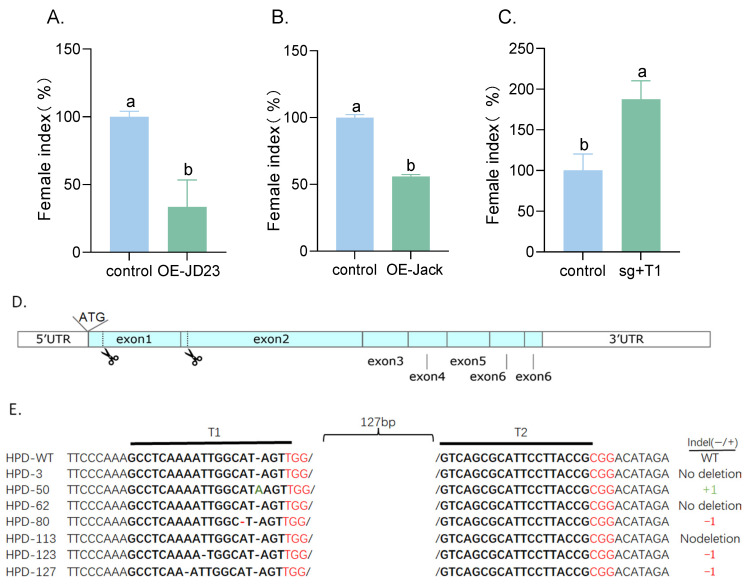
Biological assays showing effect of SCN4 on transgenic soybean lines with overexpression and knockout of *GmPDH1*. (**A**): The FIs of transgenic soybean lines were measured 25 dpi with SCN4 infection. Empty vector-transformed JD23 (SCN-susceptible) hairy roots were used as a control. OE-JD23 (*n =* 9 plants/construct examined in three independent experiments), overexpression vector-transformed JD23 (SCN-susceptible) hairy roots. (**B**): The FIs of transgenic soybean lines were measured 25 dpi with SCN4 infection. Empty vector-transformed Jack (SCN-susceptible) hairy roots were used as a control. OE-Jack *(n =* 11 plants/construct examined in three independent experiments), overexpression vector-transformed Jack (SCN-susceptible) hairy roots. (**C**): The FIs of transgenic soybean lines were measured 25 dpi with SCN4 infection. Empty vector-transformed HPD (SCN-resistant) hairy roots were used as a control. Sg + T1 (*n* = 12 plants/construct examined in at least two independent experiments), knockout vector-transformed HPD (SCN-resistant) hairy roots. Bars represent mean values of FI ± standard error. The mean values with significant differences from the control were determined by a *t*-test (*p* < 0.05) and labeled with different letters. (**D**): Gene structure and editing Schematic representation of the location of gRNA of the *GmPDH1*. (**E**): Sequences of selected fragments including Target1 and Target2. The number of nucleotides deleted (red font) and/or inserted (green font) for each plant are indicated in the columns on the right.

To investigate the regulatory role of *GmPDH1* in soybean resistance to SCN4, functional validation was performed using a heterologous expression system. The *GmPDH1* CDS was fused with a GFP reporter gene in the pCAMBIA1302 binary vector for transgenic screening (Figure 7A). Homozygous overexpression (OE) lines (OE-JD23, *n* = 9; OE-Jack, *n* = 11) were successfully generated in the SCN-susceptible cultivars JD23 and Jack. RT-qPCR analysis confirmed significant relative expression level of *GmPDH1* upregulation in transgenic lines compared to empty vector controls (EVs) (Figure 7B). To evaluate resistance, FI quantification, adapted from established protocols [46], was calculated as (transgenic line mean/wild-type control mean) × 100. Wild-type susceptibility was normalized to 100% as the baseline. SCN bioassays with SCN4 at 25 dpi revealed significant resistance enhancement. Compared to the EVs (*n =* 15), the FIs of the overexpression-positive transgenic plants of JD23 and Jack was significantly reduced (Figure 8A,B). These results confirm that *GmPDH1* mediates resistance to SCN4 in transgenic soybeans.

### 2.8. GmPDH1 Loss of Function Reduces SCN4 Resistance

To directly evaluate the functional impact of *GmPDH1* knockout on SCN resistance, the CRISPR/Cas9 system was employed to target and knockout *GmPDH1* in the transgenic hairy roots of composite HPD plants. Two guide RNA (gRNA) targets (Target1 and Target2) were designed within exons 1 and 2 of *GmPDH1* (Figure 8D). Composite soybean plants were generated by co-cultivation with *Agrobacterium rhizogenes* strain K599 harboring the CRISPR/Cas9-*GmPDH1*-gRNA1+gRNA2 construct, followed by SCN bioassays and cyst quantification at 25 dpi. In at least two independent biological replicates, CRISPR/Cas9-*GmPDH1*-gRNA1+gRNA2-transformed HPD roots (sg + T1, *n* = 12) exhibited a decrease in the FI compared to EVs (Figure 8C). The transgenic roots from each genotype or construct were collected for genotype validation. Amplification of 600 bp *GmPDH1* using sequence-specific primers confirmed CRISPR-induced mutations through Sanger sequencing, with targeted editing events verified in the HPD lines.

Next, we determined whether the target sites in transgenic hairy roots were effectively edited. Amplification primers of approximately 600 bp, including the target sites Target1 and Target2 were designed. PCR amplification was performed using these primers and the genomic DNA of positive transgenic hairy roots as the template. The amplified target bands were cut from the gel, recovered, purified, and sequenced to determine the base-editing conditions for the target sites. As shown in Figure 8E, compared with the wild type, transgenic plants had deletions and substitutions of varying degrees at Target1, indicating successful editing.

## 3. Discussion

The SCN, with its broad host range and prolonged soil persistence, poses a significant challenge for nematode control in agriculture. Cultivating resistant cultivars remains the most economically viable strategy for controlling SCN. Unraveling the molecular mechanisms underlying soybean resistance to SCN is critical for advancing breeding efforts. Although several candidate resistance genes have been functionally validated, their roles remain unexplored. This study identified a candidate genomic region (Gm:1,223,546–1,782,241) associated with SCN4 resistance through GWAS of an RIL population. Screening for high-quality non-synonymous SNPs revealed 14 candidate genes, including *Glyma.18g022500* (a known *Rhg1* component) and *GmPDH1*. RT-qPCR analyses demonstrated differential expression patterns of these genes between the resistant and susceptible cultivars across the infection stages.

The SCN completes its life cycle in approximately 30 days, with the second-stage juveniles (J2) representing the most critical phase of infection [47]. Acid fuchsin staining revealed that SCN4 successfully invaded the roots of both resistant (HPD) and susceptible (JD23) cultivars. However, significant phenotypic differences in the nematode populations emerged across the infection stages (3, 6, 9, and 12 dpi). Notably, susceptible roots harbored higher J2 larval counts at 6 dpi, whereas resistant HPD exhibited markedly fewer J3 during the J2–J3 transition phase. These observations suggest that resistance-associated genes in HPD may act between 3 and 12 dpi to impede nematode progression. In addition, this experiment revealed that the candidate genes showed differentially induced expression at 3, 6, 9, and 12 dpi of nematode development, and the difference in their expression in resistant and susceptible varieties mainly appeared in the early J2 infestation period (3 and 6 dpi), J2–J3 transition period (6 and 9 dpi), and later J3 formation stage (6, 9, and 12 dpi). These results suggest that the candidate genes may be involved in soybean defense against nematode invasion, which is consistent with the results of previous studies [49,50,51,52]. Li et al. [49] found that the relative expression level of the disease-resistant gene *GmPUB24* in HPD was upregulated 6.14 times 3 dpi than that of the uninoculated sample. Liu et al. [50] analyzed the relative expression levels of the genes *GmMOIX4* related to SCN3 resistance using RT-qPCR. The expression of *GmMOIX4* in the disease-resistant HPD variety was upregulated at 10 dpi.

Through annotation retrieval in the soybean database, the *GmPDH1* gene was found to encode the PDH enzyme, which not only participates in the Tyr synthesis as a key regulatory enzyme, but is also indirectly associated with the synthesis of other amino acids among the aromatic amino acids. After overexpressing the *GmPAL* gene, the Phe content in hairy roots increased, thereby enhancing nematode resistance. Aromatic amino acids can affect the resistance of soybean to cyst nematodes [20]. Combined with the annotation results, *GmPDH1* not only participates in the synthesis of Tyr but also shares the same synthesis precursor with Phe. Whether its function is related to the resistance against SCN remains to be investigated. Gene expression analysis indicated that, during the second-stage larval period, the resistant and susceptible varieties exhibited opposite changes in the expression levels of this gene. Moreover, the KASP markers developed using differential SNPs in the *GmPDH1* gene have been verified to be applicable for identifying resistant and susceptible individuals in natural populations. In conclusion, the *GmPDH1* gene was preliminarily screened for functional verification of candidate genes.

Analysis of the CDS sequence of the *GmPDH1* gene in disease-resistant soybean varieties in this study showed that missense mutations in the exons of resistant and susceptible soybean varieties result in amino acid polymorphisms (E151Q, V152A, and I153T) that may lead to changes in gene function. The structural properties of proteins largely determine their biological functions, and proteins with the same function have many similarities in their structures, such as similar active sites and amino acid compositions [53]. Highly conserved active sites are found in the PDH enzymes of different species that play crucial roles in the catalytic activity of the enzymes and substrate specificity. For example, site-directed mutagenesis of *E. coli* PDH and structural analysis of *Aquifex aeolicus* PDH have identified active sites, His147 and Arg250, which interact with the C4-hydroxyl group of the substrate and are essential for catalysis [24,54]. Comparative analyses revealed that the His147 active site (corresponding to His124; see the Figure S1) is present in the *GmPDH1* genes of both Williams82 and HPD, endowing these genes with catalytic activity. In addition, phylogenetic and structural analyses have revealed that the Asn222 site in *GmPDH1* is a single active site residue that determines the substrate specificity for Tyr A and Tyr sensitivity, explaining the molecular basis for PDH1 to escape from the competitive inhibition of Tyr [35]. In addition to the highly conserved sequences closely related to protein functions among different species, different soybean varieties may also have adjusted their gene functions through sequence variations during species evolution to adapt to complex environments, which may explain the results of the exon sequence changes we mentioned earlier. Certainly, the introns in the coding region and the promoter in the non-coding region are also involved in the regulation of genetic information expression and may also participate in the regulation of the functions of the *GmPDH1* gene in resistant and susceptible soybeans. In particular, multiple relevant stress response elements are present in the promoter region. Whether the promoter regulates the disease resistance function of this gene requires further investigation.

In the preliminary stage, analysis of the expression patterns of *GmPDH1* in soybean lines resistant and sensitive to SCN4 initially indicated that *GmPDH1* was related to SCN4 resistance. In the later stages, overexpressing the *GmPDH1* in SCN-sensitive soybean backgrounds and knockout of *GmPDH1* in disease-resistant soybeans provided direct evidence. When *GmPDH1* was overexpressed in the SCN-sensitive soybean varieties JD23 and Jack, the SCN4 resistance level increased significantly compared with that in the empty vector control. In addition, after knocking out *GmPDH1* in the hairy roots of the disease-resistant variety HPD, SCN resistance decreased compared with that in the wild control. In conclusion, the *GmPDH1* responds to SCN4 resistance in soybean.

The finding that the *GmPDH1* gene confers resistance to SCN4 in soybean is significant. Although a complete elucidation of the resistance mechanism is beyond the scope of this study, exploring potential pathways within the context of Tyr biosynthesis is valuable.

In legumes, both the PDH and ADH pathways contribute to Tyr synthesis. The PDH pathway initiates from chorismate, which is the end product of the shikimate pathway. CM converts chorismate to prephenate, which is subsequently dehydrogenated by PDH to form HPP, the direct precursor of Tyr. The ADH pathway involves the conversion of prephenate to arogenate by PPA-AT. Arogenate serves as a common intermediate in both Tyr and Phe biosynthesis; ADH catalyzes its conversion to Tyr, whereas alternative routes yield Phe. In *Arabidopsis thaliana*, *AT5G34930*-encoded ADH is the key enzyme regulating Tyr synthesis [32]. Soybean harbor two homologs of this gene: *Glyma.14g055300* (encoding ADH2), which mediates the plastid-localized ADH pathway, and *Glyma.18g023100* (encoding PDH1), which drives the cytosolic PDH pathway.

Subcellular localization studies by Schenck et al. [27] confirmed that *GmPDH1* operates in the cytosol and establishes a distinct Tyr biosynthetic route independent of the plastidial ADH pathway. Notably, unlike most PDH/ADH pathways, the soybean PDH pathway remains unaffected by feedback regulation [27,55,56,57]. This unique feature likely enables metabolic flux partitioning, favoring the biosynthesis of Phe, a precursor of lignin, and indirectly enhances cell wall fortification [27,58,59]. However, recent studies have shown that, in leguminous plants, the traditional ADH pathway within plastids is the main pathway for Tyr biosynthesis and cannot be compensated for by the cytosolic PDH pathway [35].

**Figure 9:**
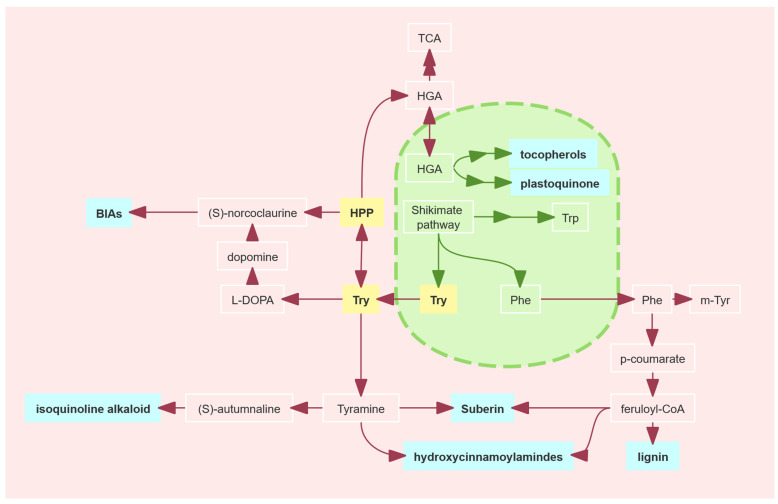
Tyrosine biosynthesis, metabolism, and catabolism. Related specialized metabolites (blue).

Tyr and its intermediate HPP serve as critical precursors for specialized metabolites vital to plant growth and defense, including antioxidant tocopherol, photosynthetic plastoquinones, and defense compounds (BIAs, hydroxycinnamoylamides and isoquinoline alkaloid) (Figure 9) [31,60,61,62,63,64,65]. While PDH activity contributes minimally to aromatic amino acid or HPP-derived metabolite accumulation under normal growth conditions, it becomes pivotal under stress by promoting the synthesis of defense-related metabolites, such as tocopherols [35].

Based on the fact that Tyr and the intermediates in the Tyr synthesis process are related to plant growth and defense, and as the PDH1 enzyme serves as a key enzyme in the Tyr synthesis process, it may indirectly regulate plant defense.

Previous studies have primarily focused on the role of *GmPDH1* in Tyr synthesis and metabolism. To the best of our knowledge, there have been no reports on the role of *GmPDH1* in SCN4 resistance. In this study, *GmPDH1* was functionally validated as a novel SCN4 resistance gene using OE and CRISPR-Cas9 knockout experiments. However, the exact mechanism—whether involving tyrosine, tyrosine (Tyr)-derived metabolites, vitamin E biosynthesis, or crosstalk with the phenylpropanoid metabolic pathway—requires further gene stacking experiments or metabolic validation. In the future, it will be necessary to further explore how PDH1 regulates stress responses by controlling tyrosine synthesis and metabolic pathways. Additionally, elucidating the roles of the other 13 unvalidated candidate genes is equally important, as they may form synergistic networks to regulate complex resistance traits.

## 4. Materials and Methods

### 4.1. Plant Materials and Growth Conditions

*Glycine max* cv. JD23 (accession number: ZDD23989) and HPD (accession number: ZDD02315), which are susceptible and resistant to SCN4, respectively, were crossed, and confirmed F1 plants were self-fertilized to F14 to develop an RIL population. In 2021 and 2022, 280 RILs and their parental lines were cultivated at the Dongyang Experimental Station of the Shanxi Academy of Agricultural Sciences for SCN resistance evaluation. The HPD landrace was used for phenotypic characterization, candidate gene cloning, and as the recipient material for genetic transformation knockout vectors. Overexpression vector transformations were performed using the JD23 and Jack backgrounds. Plants were maintained under controlled conditions: 28 °C, 16 h light/8 h dark photoperiod, and 55% relative humidity.

### 4.2. Phenotype Identification and GWAS

Cysts were artificially cultivated and collected for inoculation. First, the cysts were collected by planting susceptible varieties in diseased soil for approximately 25 days. After cyst collection using 60 mesh sifters, cysts were gently crushed in a sterile mortar with a small amount of water to release the eggs. Eggs were counted under a microscope and used to prepare an egg suspension at a concentration of 2000 eggs/mL.

In this study, an artificial inoculation method was used to characterize SCN4 resistance in soybeans. Parents and RILs were inoculated with egg suspensions, and each line was planted in five bowls, with three seedlings per bowl. White cysts attached to the roots were counted 25 dpi, and the number of cysts was recorded. Parents and RILs were categorized into different disease resistance classes based on the FI. FI < 10 was considered high resistance (HR); 10 ≤ FI < 30 was considered moderate resistance (MR); 30 ≤ FI < 60 was considered moderate susceptibility (MS); and FI ≥ 60 was considered high susceptibility (HS) [61].

$$FI=\frac{average\;number\;of\;females\;from\;differential}{average\;number\;of\;females\;from\;Lee}\times 100$$

Leaf DNA extraction, library construction, and resequencing were performed by Beijing Huada Corporation. Based on the SNPs of 280 RILs combined with the phenotypic data, GWAS was performed using the MLM in the EMMAX software. Significant SNP loci were selected, and Manhattan and Q–Q plots were plotted using the CMplot software package in R. Combined with genetic information from the soybean database and the known quantitative trait loci (QTLs), candidate genes related to SCN resistance were mined.

### 4.3. Development Process of SCN4 and Expression Patterns of Related Genes

Cyst nematodes were isolated from the susceptible varieties to obtain egg suspensions, which were then incubated in vitro. After a certain period, the second-stage larvae were collected in a 100 mL beaker and stored at 4 °C for short-term use. The susceptible variety JD23 and the disease-resistant variety HPD were selected for sodium hypochlorite acid fuchsin staining, and the number of larvae was observed and counted under a microscope.

It takes approximately 30 days for SCN4 to undergo a complete life cycle, with the second-instar larval period having the greatest influence on the effectiveness of nematode infestation. In this study, plants inoculated for 0–12 days of inoculation time were selected for interval sampling. Starting from the day of inoculation, the roots of both inoculated and uninoculated JD23 and HPD plants at 0, 3, 6, 9, and 12 days were collected as test samples, used for RNA extraction, and then transcribed into cDNA. Primer 6.0 was used to design RT-qPCR primers, and the *GmActin* gene was selected as the internal reference gene. The samples were set up in three biological replicates. The raw data were processed using Microsoft Excel (v2019, Microsoft Corporation), and the relative expression level of the genes was calculated using the 2^−ΔΔCT^ method [66]. The complete primer sequences are listed in Table S6.

### 4.4. Development and Validation of KASP Markers for the SCN4 Resistance Gene GmPDH1 in Glycine Max

Candidate SNP M0526, located at positions 1, 680, 376 on chromosome 18, associated with SCN, was identified through association analysis. Primers were designed using the *Glycine max* cv. Williams 82 reference genome (Glycine_max_v4.0) with DNAMAN (v9) and Primer3 Plus (v4.1.0), comprising three primers per set: two fluorescence-labeled forward primers (F-Fam/F-Hex) and one universal reverse primer (complete primer sequences are listed in Table S7).

The KASP reaction test was performed using the AQPTM genotyping system, also known as an Allele-Specific Quantitative PCR based genotyping assay (AQP). Marker validation involved 264 advanced-generation RILs derived from HPD × JD23 and 96 natural population materials.

### 4.5. Cloning and Bioinformatics Analysis of GmPDH1

*GmPDH1* genomic/CDS/amino acid sequences were retrieved from Phytozome (v13, https://phytozome.jgi.doe.gov/ (accessed on 1 August 2023)) for full-length CDS amplification. Sequence alignments were performed using SnapGene (v6.1.2). Protein physicochemical properties, including the isoelectric point and molecular weight, were calculated using ExPASy ProtParam (https://expasy.org/ (accessed on 1 August 2023)). Secondary structure predictions were performed using SPOMA and SWISS-MODEL with default parameters. The primers used for CDS amplification are listed in Table S6.

### 4.6. GmPDH1 Expression Analysis

HPD cDNA served as a template for PCR amplification. Full-length primers were designed based on the published CDS of *GmPDH1*, incorporating 15–20 bp homologous arms and restriction sites (*Nco* I/*Spe* I) at both termini. The overexpression vector, pCAMBIA1302, was subjected to double digestion with *Nco* I and *Spe* I (NEB, Beijing, China). Digestion efficiency was verified by 1% agarose gel electrophoresis, followed by gel extraction and purification using a TIANgel purification kit (Tiangen, Beijing, China). Purified PCR products and linearized vectors were recombined using the ClonExpress II One Step Cloning Kit (Vayme, Nanjing, China), following the manufacturer’s protocol [67]. Recombinant plasmids were transformed into *E. coli* DH5α and selected on LB agar containing 50 μg/mL kanamycin. Plasmid DNA was isolated using a TIANprep Mini Plasmid Kit (Tiangen, Beijing, China) and sequenced (Sangon Biotech, Shanghai, China). The plasmid with the correct sequencing was transformed into *A. rhizogenes* strain K599 for genetic transformation. Transformed colonies were selected on LB medium supplemented with 50 μg/mL rifampicin and 100 μg/mL kanamycin. The primers used to construct the OE vectors are listed in Table S6.

### 4.7. GmPDH1 CRISPR gRNA Design and Off-Target Analysis

The pCBC-DT1T2 and pHSE401 vector systems for gene editing were kindly provided by Qijun Chen’s group at China Agricultural University. First, the CRISPR/Cas9 target website (http://crispr.tefor.net/ (accessed on 15 August 2023)) was used to design the targets. The CDS of *GmPDH1* was used as the target sequence to design gRNA targets. The specific target sequence is shown in Table S6. For construction of the gene-editing vectors, the method described by Xing et al. was used [68]. The zymosynthesis product was transformed into the competent cells of *E. coli* DH5α. After screening the transformed cells with kanamycin and spectinomycin and performing PCR characterization, positive monoclonal colonies were selected, and their plasmids were extracted for sequencing. Plasmids with the correct sequencing results were then transferred into *A. rhizogenes* strain K599 for genetic transformation.

### 4.8. Evaluation of SCN4 Resistance of Transgenic Hairy Roots

Using the *A. rhizogenes*-mediated one-step transformation method [69], JD23 and HPD were genetically transformed. Seedlings were taken from 7 days old aseptically germinated batch after chlorine sterilization. Their original roots were cut off, and the trauma was cut out with a sterile scalpel at the hypocotyls of seedlings at 0.7–1 cm at an angle of 45°, dipped in the recombinant strain, inserted into moist, sterile vermiculite, and placed in a 25 °C, high humidity (55–80%), relatively sterile environment for 14–20 days before washing the root system for positive plant identification.

The disease-resistant variety HPD, susceptible variety JD23, and wild-type hairy roots of Jack were selected as control groups. The OE vector pCAMBIA1302-*GmPDH1* was transformed into the susceptible varieties JD23 and Jack, and the knockout vector CRISPR/Cas9-*GmPDH1*-gRNA1+gRNA2 construct was transformed into HPD. An egg suspension of SCN4 was inoculated, and after culturing for 25 days, the total number of cysts in the control and treatment groups was counted.

### 4.9. Statistical Analysis

The FI and relative gene expression levels in different samples were presented as mean ± standard deviations of at least three replications. SPSS (v22.0, IBM corporation, Armonk, NY, USA) was used to analyze the difference significance of measured parameters among different samples at the *p* < 0.05. GraphPad Prism (v9) was used for the figure drawing.

## Data Availability

The original contributions presented in this study are included in the article. Further inquiries can be directed to the corresponding author.

## Supplementary Materials

The following supporting information can be downloaded from: https://www.mdpi.com/article/10.3390/plants14121877/s1, Table S1: Basic descriptive statistics of FI value in multiple environment; Table S2: Genes that is missense-variant in different varieties at high frequency locus; Table S3: Developmental dynamics of Heterodera glycines in soybean roots; Table S4: Cis-acting elements existed in the promoters of GmPDH1 gene; Table S5: Physiological and biochemical properties of GmPDH1 proteins*; Figure S1: Clustal alignment of the GmPDH1 protein; Table S6: Primers used for gene cloning, vector construction, and RT-qPCR; Table S7: Primers used for the KASP assay of SCN4 resistance-associated *GmPDH1* locus.

## Abbreviations

The following abbreviations are used in this manuscript:

SCN	Soybean cyst nematode
RIL	Recombinant inbred line
HPD	Huipizhiheidou
JD23	Jindou23
GWAS	Genome-wide association analysis
RT-qPCR	Reverse transcription quantitative PCR
KASP	Kompetitive Allele Specific PCR
NSF	N-ethylmaleimide-sensitive fusion protein
Tyr	L-tyrosine
CM	Chorismate mutase
PPA-AT	Prephenate aminotransferase
PDH	Prephenate dehydrogenase
ADH	Alcohol dehydrogenase
Phe	L-Phenylalanine
HPP	4-hydroxyphenylpyruvic acid
DPI	Days post-inoculation
CDS	Coding sequence
FI	female index
gRNA	Guide RNA
SNP	Single nucleotide polymorphism
MLM	Mixed linear model
AQP	Allele-Specific Quantitative PCR
OE	Overexpression
EVs	Empty vector controls
