# Supplementary material for: Gene Localization and Functional Validation of GmPDH1 in Soybean Against Cyst Nematode Race 4
Source: Plants (Basel). 2025 Jun 19;14(12):1877. doi: 10.3390/plants14121877 (PMC12197188; doi:10.3390/plants14121877)
Supplement: Supplementary file 1 [file plants-14-01877-s001.zip › plants-3635049-supplementary.pdf]

Table S1 Basic descriptive statistics of FI value in multiple environment

	Parent		RIL population				
Environment	HPD	JD23	Mean±SD	Range of variation	CV/%	Kurtosis	Skewmess
E1	20.28±3.2	63.82±12.24	86.39±35.29	2.13~177.28	40.85	-0.06	-0.37
E2	16.51±2.62	85.28±13.02	79.21±36.05	4.59~216.64	45.51	1.59	0.687

Note: E1 and E2 indicate the identification of disease resistance in different environments.

Table S2 Genes that is missense-variant in different varieties at high frequency locus

Soybean candidate gene	Start	End	Gene ID	annotion
Glyma.18g017500	1243660	1244609	AT5G65030.1	C2 domain-containing protein
Glyma.18g017600	1247169	1262072	AT4G39850.1	ATP-binding ABC transporter
Glyma.18g017900	1294709	1297852	AT5G65160.1	tetratricopeptide repeat (TPR)-containing protein
Glyma.18g021300	1560766	1564062	AT2G40480.1	Plant protein of unknown function (DUF827)
Glyma.18g021500	1576574	1580301	AT2G40490.1	Uroporphyrinogen decarboxylase
Glyma.18g021800	1679597	1682003	AT5G05340.1	response to oxidative stress
Glyma.18g023000	1639181	1643814	AT2G13620.1	Sodium/hydrogen exchanger family
Glyma.18g023100	1669700	1673686	AT5G34930.1	Prephenate dehydrogenase
Glyma.18g023200	1682237	1689587	AT2G40400.2	Protein RETICULATA-related
Glyma.18g022500	1604175	1606426	AT3G56190.1	Soluble NSF attachment protein, SNAP

Table S3 Developmental dynamics of *Heterodera glycines* in soybean roots

dpi	Stage	Resistanct（count/plant）	Susceptible (counts/plant)
6	J2	364	877
10	J2	395	505
14	J3	58	132
18	J4♀/♂	18♀/♂	24♀/12♂
26	Cysts	0	60

Table S4 Cis-acting elements existed in the promoters of *GmPDH1* gene

Element name	sequence	Response
ABRE	ACGTG	Abscisic acid responsive element
Box 4	ATTAAT	Light responsive element
CGTCA-motif	CGTCA	MeJA responsive element
TGACG-motif	TGACG	MeJA responsive element
ARE	AAACCA	anaerobic induction element
G-box	CACGAC	light responsive element
MRE	AACCTAA	MYB binding site involved in light responsiveness
CAT-box	GCCACT	meristem-specific regulatory elements
p-box	CCTTTTG	gibberellin-responsive element
TC-rich repeats	GTTTTCTTAC	defense and stress responsive element

Table S5 Physiological and biochemical properties of GmPDH1 proteins*

Gene	CDS	Amino acids	MW (kDa)	pI	GRAVY	Instability	Aliphatic index
GmPDH1	816	271	30.60	6.55	-0.226	37.85	81.29

*CDS, coding sequence; MW, molecular weight of the amino acid sequence; pI, theoretical isoelectric point; GRAVY, grand average of hydropathicity

Table S6 Primers used for gene cloning, vector construction, and RT-qPCR

Gene	Primer sequence
**RT-qPCR**	
GmActin-F	AAGCTGTTCTCTCCTTGTACGCC
GmActin-R	GCACAGTGTGAGACACACCATCA
Glyma.18g017500-F	CATGTGGTGGGAAAGGGTGAGATTC
Glyma.18g017500-R	CACTTGTACTTGTGGTGCCTTGAAC
Glyma.18g017600-F	TTCACGCACGATTAAGGACTCACTC
Glyma.18g017600-R	CCTGAATCATGCCAAACCACCAATG
Glyma.18g017901-F	GAGGCAATTCACCAGAGAGGAAACC
Glyma.18g017901-R	AATTGTTGGCTACGGGAGAAGAACC
Glyma.18g017902-F	GAGGCAATTCACCAGAGAGGAAACC
Glyma.18g017902-R	AATTGTTGGCTACGGGAGAAGAACC
Glyma.18g021301-F	TGCTGCTAAGGAGGCTCTAAGGAG
Glyma.18g021301-R	TGCTGTTGTTGACCTGGTTACATCC
Glyma.18g021302-F	TGCTGCTAAGGAGGCTCTAAGGAG
Glyma.18g021302-R	TGCTGTTGTTGACCTGGTTACATCC
Glyma.18g021500-F	GCGTCAACACTGCCTTCACCTC
Glyma.18g021500-R	TTGGTTCTGCAACTGTTCCTTGGAG
Glyma.18g022500-F	TGCTGAATTGTACGAGGGTGAACAG
Glyma.18g022500-R	GCATTGGTTCGCAGAAGTTGTCAC
Glyma.18g023001-F	CACCGAGGCAACCCATTAGAATCC
Glyma.18g023001-R	TGGCTTGAGACATGCGTCCATTATC
Glyma.18g023002-F	GGACGCATGTCTCAAGCCACTAG
Glyma.18g023002-R	TTCAAGCACTACAGCACCCTTCAC
Glyma.18g023003-F	GCCTCCTTCTGTCCGTAGTTGC
Glyma.18g023003-R	CATTAACCGAACCGCCACCTGAG
Glyma.18g023100-F	GTCAACCTCATCCTCTTCCCAAAGC
Glyma.18g023100-R	CGAGAAGTTGCTGTGAGAGTGTGG
Glyma.18g021800-F	CGCTCGAGACTCTGTTGTTTA
Glyma.18g021800-R	AGGCATCAATGTCGGAATCA
Glyma.18g023200-F	GTCTCAAGCCACTAGGTCAATC
Glyma.18g023200-R	CAGCACCCTTCACAGGAAATA
**Cloning**	
GmPDH1-F	CAAATTGGCAGGGAAAATCCTCT
GmPDH1-R	TCGTCCTTTGTATCATCAGCGTT
**Overexpression (OE)**	
GmPDH1-F	acgggggactcttgaccatgAGGGATGCTTCTGACATCACAAT
GmPDH1-R	aagttcttctcctttactagtTTTGTCCTTTTTGATCATCAGCG
GFP-F	GTTGTCCCAATTCTTGTTGAATTAGATGG
GFP-R	GTTACAAACTCAAGAAGGACC
**CRISPR/Cas9**	
GmPDH1-DT1-BsF-gRNA1	ATATATGGTCTCGATTGGCCTCAAAATTGGCATAGTGTT
GmPDH1-DT1-F0-gRNA1	TGGCCTCAAAATTGGCATAGTGTTTTAGAGCTAGAAATAGC

Gene	Primer sequence
GmPDH1-DT2-R0-gRNA2	AACCGGTAAGGAATGCGCTGACCAATCTCTTAGTCGACTCTAC
GmPDH1-DT2-BsR-gRNA2	ATTATTGGTCTCGAAACCGGTAAGGAATGCGCTGACC
U626-IDF	TGTCCCAGGATTAGAATGATTAGGC
U629-IDF	TTAATCCAAACTACTGCAGCCTGAC
U629-IDR	AGCCCTCTTCTTTCGATCCATCAAC
**CRISPR/Cas9 sequencing**	
GmPDH1-gRNA-Target-F	CCAAATTGGCAGGGAAAATCC
GmPDH1-gRNA-Target-R	AATTGGCTCTTAGCAGCTGC
**RT-qPCR OE**	
GmPDH1-OE-F	GGACGCATGTCTCAAGCCACTAG
GmPDH1-OE-R	TTCAAGCACTACAGCACCCTTCAC

Table S7 Primers used for the KASP assay of SCN4 resistance-associated *GmPDH1* locus

Markers	Sequence of KASP markers (5`- 3`)	Tm	Product length
M0526 FAM	GTATGACAAAGTTCGGATAAGAGACg	58℃	96bp
M0526 HEX	GTATGACAAAGTTCGGATAAGAGACc	58℃	96bp
M0526 Common R	TGGGTATTGACAAAGGATATTAACC	58℃	96bp

Figure S1 Clustal alignment of the GmPDH1 protein

```
Williams82-PDH1    ........................................     0
HPD-PDH1           ........................................     0
Eco-tyA            MVAELTALRDCIDEVDKALLNLLAKRLELVAEVGEVKSRF    40
A.aeolicus-VF5     ........................................     0
Consensus

Williams82-PDH1    ..................................MSTSSS     6
HPD-PDH1           ..................................MSTSSS     6
Eco-tyA            GLPIYVPEREASMLASRRAEAEALGVPPDLIEDVLRRVMR    80
A.aeolicus-VF5     ..........MAILSSMFNPSPPQGFCKKNIIKILKSLSM    30
Consensus

Williams82-PDH1    SQSLKIGIVGFG.NFGQFLAKTMIKQGHTLTATSRSDYSE    45
HPD-PDH1           SQSLKIGIVGFG.NFGQFLAKTMIKQGHTLTATSRSDYSE    45
Eco-tyA            ESYSSENDKGFK.TLCPSLRPVVIVGGGCMGRLFEKMLT   119
A.aeolicus-VF5     QNVLIVGVGFMGGSFAKSLRRSGFKGKIYGYDINPESISK    70
Consensus                            l

Williams82-PDH1    LC...LQMGIHFFRDVSAFLTADIDVIVLCTSILSLSEVV    82
HPD-PDH1           LC...LQMGIHFFRDVSAFLTADIDVIVLCTSILSLSEVV    82
Eco-tyA            LSGYQVRILEQHCWDRAADIVADAGMVIVSVPIHVTEQVI   159
A.aeolicus-VF5     AVD..LGIIDEGTTSIAKVEDFSPDFVMLSSPVRTFREIA   108
Consensus

Williams82-PDH1    GSMPLTSLKRPTLFVDVLSVKEHPRELLLRELPEDSDILC   122
HPD-PDH1           GSMPLTSLKRPTLFVDVLSVKEHPRELLLRELPEDSDILC   122
Eco-tyA            GKLPP..LPKDCILVDLASVKNGP..LCAMLVAHDGPVLG   195
A.aeolicus-VF5     KKLSY.ILSEDATVTDQGSVKGKL..VYDLENILGKRFVG   145
Consensus                 l       d  svk

Williams82-PDH1    THPMFGPQ......TAKNGWTDHTFMYDKVRIRDEVICSN   156
HPD-PDH1           THPMFGPQ......TAKNGWTDHTFMYDKVRIRDQATCSN   156
Eco-tyA            LHPMFGPD......SGS..LAKQVVVWCDG..RKPEAYQW   225
A.aeolicus-VF5     GHPIAGTEKSGVEYSLDNLYEGKKVILTPTKKTDKKRLKL   185
Consensus           hp  g

Williams82-PDH1    FIQIFATEGCKMVQMSCEEHDR....AAAKSQFITHTIGR   192
HPD-PDH1           FIQIFATEGCKMVQMSCEEHDR....AAAKSQFITHTIGR   192
Eco-tyA            FLEQIQVWGARLHRISAVEHDQNMAFIQALRHFATFAYGL   265
A.aeolicus-VF5     VKRVWEDVGGVVEYMSPELHDYVFGVVSHLPHAVAFALVD   225
Consensus                  g      s   hd

Williams82-PDH1    TLGEMDIQSTPIDTKG...FETLVKLKETMMRNSFDLYSG   229
HPD-PDH1           TLGEMDIQSTPIDTKG...FETLVKLKETMMRNSFDLYSG   229
Eco-tyA            HLAEENVQLEQLLALSSPIYRLELAMVQRLFAQEPQLYAD   305
A.aeolicus-VF5     TLIHMSTPEVDLFKYP....GGGFKDFTRIAKSDPIMWRD   261
Consensus           l

Williams82-PDH1    LFVYN........RFARQELENLEHALHKVKETLMIQRTN   261
HPD-PDH1           LFVYN........RFARQELENLEHALHKVKETLMIKKDK   261
Eco-tyA            IIMSSERNLALIKRYYKRFGEAIELLEQGDKQAFIDSFRK   345
A.aeolicus-VF5     IFLEN.......KENVMKAIEGFEKSLNHLKELIVREAEE   294
Consensus                              e  e      k

Williams82-PDH1    GEQGHKRTES.................                271
HPD-PDH1           GEQGHKRTES.................                271
Eco-tyA            VEHWFGDYAQRFQSESRVLLRQANDNR                372
A.aeolicus-VF5     ELVEYLKEVKIKRMEID..........                311
Consensus
```
